# Influence of eye movements on academic performance: A bibliometric and citation network analysis

**DOI:** 10.16910/jemr.15.4.4

**Published:** 2022-09-07

**Authors:** Adrián Salgado-Fernández, Ana Vázquez-Amor, Cristina Alvarez-Peregrin, Clara Martinez-Perez, Cesar Villa-Collar, Miguel Ángel Sánchez-Tena

**Affiliations:** Adrián Salgado Óptica y Audición, Spain; Universidad Europea de Madrid - Faculty of Biomedical and Health Science, Spain; ISEC LISBOA - Instituto Superior de Educação e Ciê ncias, Portugal; Department of Optometry and Vision, Faculty of Optics and Optometry, Universidad Complutense de Madrid, Spain

**Keywords:** oculomotor, motor skills, citation network, eye movements

## Abstract

Background: For many years it has been studied how eye movements influence reading and learning ability. The objective of this study is to determine the relationships between the different publications and authors. As well as to identify the different areas of research ocular movement.; Methods: Web of Science was the database for the search of publications for the period 1900 to May 2021, using the terms: “Eye movement" AND “Academic achiev*”. The analysis of the publication was performed using the CitNetExplorer, VOSviewer and CiteSpace software.; Results: 4391 publications and 11033 citation networks were found. The year with the most publications is 2018, a total of 318 publications and 10 citation networks. The most cited publication was "Saccade target selection and object recognition: evidence for a common attentional mechanism." published by Deubel et al. in 1999, with a citation index of 214. Using the Clustering function, nine groups were found that cover the main research areas in this field: neurological, age, perceptual attention, visual disturbances, sports, driving, sleep, vision therapy and academic performance.; Conclusion: Even being a multidisciplinary field of study, the topic with the most publications to date is the visual search procedure at the neurological level.

## Introduction

Ocular motility has been studied since the 20th century (16); therefore, the first era on the research of eye movements dates from 1879 to 1920 (51). In recent years, as stated by Bilbao & Piñero (7), researchers have developed a great interest about this topic. Within eye movement, those which are present while reading must be highlighted, tracking movements, saccadic movements, regressions and solidity during fixation, since this is a key process in the learning development of school-age children (7).

During reading processes, the eyes move through the text making a series of saccadic movements with different ranges and directions as well as different fixations with variable durations. These movements tend to go in a forward direction, that is to say, the eyes fixate on one word before moving onto another. However, in order to fixate on a previous word, or to move to the next line of the text, a backwards movement is performed (regression). Saccadic movements are the fastest movement that the human body is capable of performing, with an average speed of 100° to 800° per second and a frequency of 100,000 saccades per day (51).

The necessary skills, which are most influenced by vision, to achieve a good academic performance are: a fluent reading, precision and a good understanding of the text. These skills depend on eye movements (37; Faber et al., 2020). The tracking eye movement is considered as a tool that can be used to investigate, in real-time, the cognitive processes that are involved in reading. Studies have shown that readers with faster reading speeds tend to have fewer and shorter fixations, larger saccadic movements, fewer regressions and more extended perception periods when reading sentences and texts (52; 22; 17; 24). On the other hand, highly able readers presented with short fixations and fewer regressions than average readers (2; 33). A recent study conducted by Hindmarsh et al. (29), found that children with an average or higher reading ability had better control of their vertical and horizontal eye movements and they also made a greater number of eye movements between the lines than those with lower reading abilities.

Eye movements have many functions in a range of everyday tasks, given that these work in coordination with body and head movements. In social interactions, eye movements provide us with information (i.e.: by looking at someone´s face or following their look) and indicate that we are looking at someone in particular. In other words, eye movements serve an additional purpose as a communicative signal. On the other hand, for example while driving, the interaction between head and eye movements is complex, therefore meaning that saccadic movements, smooth pursuit vergence eye movements and the vestibulo-ocular reflex are required (20; 64; 35).

Citation network analysis is a powerful tool, which allows us to analyse, classify and deepen the scientific literature on a specific subject. Moreover, through this type of analysis is it possible to determine the most cited article and create groups that allow for links between articles and authors to be established. This therefore means that it is a great tool for broadening our knowledge on a specific field of interest (23).

This study presents an analysis of citation networks. In which the relationship between authors and publications is analysed. As well as an in-depth analysis of the research areas with the greatest interest within the research field of the influence of eye movements on academic performance.

## Methods

### Database

The search of publications was carried out in the Web of Science (WOS) database, using the following search terms: "Eye movement" AND “Academic achiev*”. These terms were used in accordance with the aim of this study, how eye movements influence academic performance.

As the search results had articles in common, the Boolean operator “NOT” was used, as well as the truncation symbol “*”, which was used to search for the singular and plural forms of the terms. Therefore, in the second search we used the following terms: (“Eye movement*” AND “Academic performance” NOT “Academic achiev*”), in the third search we used the following terms: (“Eye movement*” AND “Binocular vision” NOT “Academic performance” NOT “Academic achiev*”), and in the fourth search, the following terms: (“Eye movement*” AND “Visual performance*” NOT “Binocular vision” NOT “Academic performance” NOT “Academic achiev*”). The selected time interval to carry out the search was from 1900 to May 2021.

### Data analysis

Once the Web of Science bibliography is downloaded by exporting plain text files, it is loaded into the *CitNetExplorer* software. CitNetExplorer software [(Centre for Science and Technology Studies), Leiden, The Netherlands] has been used for the analysis of publications and the creation of citation networks.

Using the Citation Score attribute, the quantitative analysis was performed, in order to quantify the internal connections of the Web of sciences database and other external databases (Current Contents Connect, Data Citation Index, Derwent Innovations Index, KCI-Korean Journal Database, Medline, Russian Science Citation Index, ScIELO Citation Index).

First, the clustering function has been used, which is based on the formula developed by VanEck in 2012 (62), where c_i_ denotes the cluster to which node *i* is assigned, *δ*(c_i_ , c_j_) denotes a function that equals 1 if ci = cj and 0 otherwise, and γ denotes a resolution parameter that determines the level of detail of the clustering. The higher the value of γ, the larger the number of clusters that will be obtained. This formula allows to obtain the connections between the publications

$$V\left(c_{1\;},\ldots ,c_{n}\right)=\sum_{i<j}^{}\delta \;(c_{i},c_{j})(s_{\text{ij}}-\gamma )$$

Secondly, the "core Publications" function has been used, which consists of identifying the publications that are considered to be at the core of a citation network. Only publications with 4 or more citations were considered.

For the use of The CiteSpace software (5.6.R2), the bibliography downloaded from the Web of Science is also used. First, the period of years to be analyzed is selected. Next, it is selected based on what you want to analyse, the country, the institutions, authors, keyword, etc.; and thus obtain the following parameters: *H Index* (number and level of scientific production of authors and institutions). *Degree* (number of connections between authors, institutions, countries, etc.). *Centrality* (Determines the importance of the nodes in the research cooperation network, and the half-life is a parameter that represents the continuity of institutional research from a temporal perspective).

VOSviewer software allows the visualization and creation of bibliometric networks. Therefore, it was used for creating the graphs. To obtain the graphs, the CitNetexplorer software groups are downloaded in the Pakej format. Next, this file is loaded in Vosviewer in the Create- Create a map based on network data section

On the other hand, the Web of Science and Scimago Journal & Country Rank databases have been used to obtain bibliometric data and the impact of the journals.

## Results

The first articles on eye movements were published in 1976. The period of study was from 1900 to May 2021. Through the WOS search, 4391 publications and 11033 citation networks were found.

**Figure 1.**
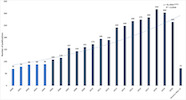
Number of publications per year

As shown in Figure 1, the number of publications on eye movements increased exponentially since 2005, more than 100 publications per year, (1976-2004: 19.7% of publications; 2005-2021:80.2% of publications). 2018 was the year with the highest number of publications: 318 publications and 10 citation networks.

Table 1 shows the 20 most cited publications within this citation network. The most cited article was written by Deubel et al. (15), which was published in 1998 and has a citation index of 213. This article analysed the spatial interaction and the saccadic movements of the eyes. Therefore, the data showed that visual discrimination is better when the discrimination stimuli and the saccadic movement move toward the same object. That is to say, it is difficult to direct the attention to the object that is being discriminated whilst the saccadic movement moves towards another close object. In conclusion, the obtained data highlighted the importance of a compulsory and selective link between the saccadic programming and the visual attention towards an object in common.

After having analysed the 20 most cited articles, 10 of them concern the visual search procedure in primates on a neurological level (32; 44; 30; 3; 61; 12; 49; 6; 45; 26), 3 of them addressed the neuronal mechanisms that generate the saccadic movements and how these vary with age (43; 18; 4), 3 addressed the association between saccadic movements and the perceptive attention (15; 39; 42), 3 considered the importance of ocular-motor movements within the sports field (54; 21; 66), and one of them was about ocular-motor movements in patients with alterations in their field of vision (36).

**Table 1.** 20 most cited publications

**Author**	**Title**	**Journal**	**Year**	**Citation Index**	**Links**
Deubel et al.	Saccade target selection and object recognition: evidence for a common attentional mechanism.	Vision Res. 1996; 36(12):1827-37.	1998	213	137
Koweler et al.	The role of attention in the programming of saccades	Vision Res. 1995; 35(13):1897-916	1995	168	121
Itti et al.	A saliency-based search mechanism for overt and covert shifts of visual attention	Vision Res. 2000; 40(10-12):1489-506.	2000	147	81
Najemnik et al.	Optimal eye movement strategies in visual search	Nature. 2005; 434(7031):387-91.	2005	84	69
Munoz et al.	Age-related performance of human subjects on saccadic eye movement tasks	Exp Brain Res. 1998; 121(4):391-400	1998	77	41
Hollingworth et al.	Accurate visual memory for previously attended objects in natural scenes	J. Exp. Psychol. Hum. Percept. Perform. 2002; 28(1), 113–136.	2002	58	41
Ballard et al.	Memory Representations in Natural Tasks	J Cogn Neurosci.1995; 7(1):66-80	1995	54	34
Tatler et al.	Eye guidance in natural vision: Reinterpreting salience	J Vis. 2011; 11(5):5.	2011	52	43
Everling et al.	The antisaccade: a review of basic research and clinical studies	Neuropsychologia. 1998;36(9):885-99.	1998	51	33
Moore et al.	Control of eye movements and spatial attention	Proc Natl Acad Sci U S A. 2001;98(3):1273-6.	2001	51	36
Savelsbergh et al.	Visual search, anticipation and expertise in soccer goalkeepers	J Sports Sci. 2002 Mar;20(3):279-87.	2002	48	33
Borji et al.	State-of-the-Art in Visual Attention Modeling	IEEE Trans Pattern Anal Mach Intell. 2013;35(1):185-207.	2013	48	23
Rao et al.	Eye movements in iconic visual search	Vision Res. 2002; 42(11):1447-63.	2002	46	39
Konstantopoulos et al.	Driver's visual attention as a function of driving experience and visibility. Using a driving simulator to explore drivers’ eye movements in day, night and rain driving	Accid Anal Prev. 2010; 42(3):827-34.	2010	44	15
Bertera et al.	Eye movements and the span of the effective stimulus in visual search	Percept Psychophys. 2000;62(3):576-85.	2000	41	32
Navalpakkam et al.	Modeling the influence of task on attention	Vision Res. 2005 Jan;45(2):205-31.	2005	40	28
Gegenfurtner et al.	Expertise Differences in the Comprehension of Visualizations: a Meta-Analysis of Eye-Tracking Research in Professional Domains	Educ Psychol Rev.2011; 23, 523–552.	2011	39	33
Williams et al.	Visual Search Strategy, Selective Attention, and Expertise in Soccer	Res Q Exerc Sport. 1998;69(2):111-28	1998	38	27
Hayhoe et al.	Visual memory and motor planning in a natural task	J Vis. 2003;3(1):49-63.	2003	38	26
Barnes et al.	Cognitive processes involved in smooth pursuit eye movements	Brain Cogn. 2008; 68(3):309-26.	2008	38	31

### Description of the publications

**Figure 2.**
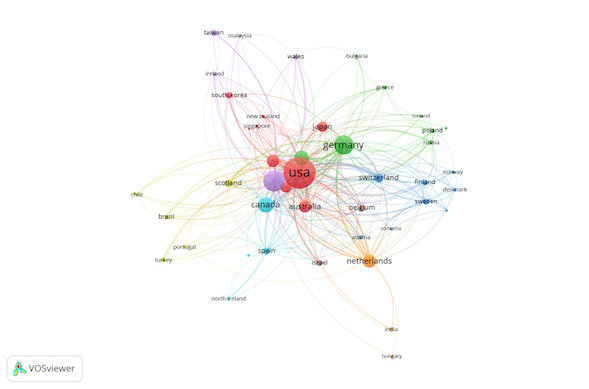
Collaboration amongst countries

Of all publications, 84.8% were articles, 6.1% were proceedings papers, 5.9% were reviews, and the remaining 3.2% were meeting abstracts, book chapter or editorial materials. With regards to the language of the publications, 99% were in English, 0.8% were in German and the remaining 0.2% were in Russian. As shown in Figure 2 and Table 2, the countries with the highest number of publications were the United States (33.9%), England (14.9%) and Germany (13.1%). Figure 2 shows the collaboration amongst countries, as well as the group they belong to. The colour of an article represents the group they belong to and the lines among elements represent their strength.

Table 2 shows the main characteristics of the 5 most important groups in Figure 2.

**Table 2.** Characteristics of the main countries

**Group**	**Colour**	**Main countries**	**Publications**	**Centrality**	**Degree**	**Half-life**	**Connections**
1°	Red	USA	1492	0.55	57	34.5	519
2°	Green	Germany	574	0.17	39	21.5	355
3°	Blue	Switzerland	129	0.06	21	17.5	98
4°	Yellow	Scotland	82	0.06	23	13.5	92
5°	Violet	England	655	0.44	53	21.5	414

The research area on eye movements is multidisciplinary. It is worth noting the field of Psychology (34.7%) and Neuroscience (34.2%) (Table 3).

The institutions with the highest number of publications (Table 4) were University of Toronto (1.8%), New York University (1.5%) and University of Tubingen (1.5%). Table 5 shows the main journals and the number of publications that have published on eye movements.

The most commonly used keywords were “Eye-movements”, “Attention”, “Performance”, “Saccadic Eye movements” and “Perception”.

Table 6 shows the most used keywords in the most relevant publications.

**Table 3.** Top 10 research areas with the highest number of publications

**Category**	**Frequency**	**Centrality**	**Degree**	**Half-life**
Psychology	1525	0.11	57	21.5
Neurosciences & Neurology	1502	0.15	63	18.5
Neurosciences	1369	0.11	52	17.5
Psychology, Experimental	832	0.01	29	21.5
Ophthalmology	808	0.02	23	34.5
Engineering	443	0.29	84	24.5
Computer Science	427	0.20	74	23.5
Clinical Neurology	253	0.08	41	19.5
Science &Technology-Other Topics	231	0.03	20	24.5
Behavioural Sciences	230	0.05	34	18.5

**Table 4.** Top 10 institutions with the highest number of publications

**Category**	**Frequency**	**Centrality**	**Degree**	**Half-life**	**Connections**
University of Toronto	78	0.00	29	13.5	531
New York University	68	0.00	31	24.5	675
University of Tubingen	68	0.00	16	15.5	617
Harvard University	64	0.00	38	9.5	646
University College of London	56	0.00	37	6.5	368
University of Utrecht	53	0.00	13	13.5	454
University of Illinois	50	0.00	16	9.5	525
University of Munich	48	0.00	22	7.5	899
University California Berkeley	48	0.00	19	17.5	245
Le Centre national de la recherche scientifique	47	0.00	21	14.5	345

**Table 5.** Top 20 journals with the most publications

**Journal**	**Total publications**	**Impact Factor (2020)**	**Quartile Score**	**SJR (2020)**	**Citations/Docs (2 years)**	**Total Citations (2020)**	**H Index**	**Country**
Journal of vision (open access- CC BY or a CC BY-NC-ND license)	214	2.15	Q3	1.126	3.838	2855	113	United States
Vision Research	213	2.82	Q3	1.127	3.744	2459	164	United Kingdom
Journal of neurophysiology	126	2.71	Q3	1.302	5.234	7092	245	United States
Experimental brain research	121	1.97	Q4	0.782	3.733	3762	172	Germany
Plos One (open access- CC BY license)	120	3.79	Q2	0.99	6.222	379308	332	United States
Journal of Neuroscience	85	6.99	Q1	3.483	11.210	32012	455	United States
Neuropsychologia	85	3.56	Q2	1.439	3.033	3228	206	England
Investigative Ophthalmology & Visual Science (open access- CC BY or a CC BY-NC-ND license)	67	4.85	Q1	1.935	8.22	17287	218	United States
Attention Perception & Psychophysics	65	2.31	Q3	1.151	3.748	2321	116	United States
Journal of Experimental Psychology: Applied	57	2.96	Q2	1.004	4.448	881	84	United States
Optometry and Vision Science	54	1.97	Q3	0.779	1.522	769	97	United States
Frontiers in Psychology (open access- CC-BY license)	48	3.62	Q2	0.947	2.782	24199	110	Switzerland
Visual Cognition	46	1.89	Q3	0.797	1.365	270	81	United Kingdom
Perception	45	1.78	Q4	0.619	1.192	330	91	United States
Scientific Reports (open access- CC-BY license)	40	5.13	Q1	1.240	4.130	282734	213	United Kingdom
Journal of eye Movement Research (open access- CC-BY license)	40	1.25	Q4	0.250	1.124	142	20	Switzerland
Transportation Research part f-traffic Psychology and Behaviour	38	3.78	Q2	1.231	3.903	3204	94	United Kingdom
Frontiers in Human Neuroscience (open access- CC-BY license)	33	3.98	Q3	1.129	3.154	5657	114	Switzerland
Acta Psychologica	32	2.07	Q4	0.865	1.656	850	97	Netherlands
Cognition	28	4.33	Q1	2.080	3.549	2744	187	Netherlands

**Table 6.** Top 20 most used keywords

**Keyword**	**Frequency**	**Degree**	**Total link strength**
Eye-movements	1444	68	14039
Attention	859	96	6935
Performance	781	117	5640
Saccadic Eye movements	755	106	5944
Perception	511	91	3997
Saccades	365	116	3188
Information	349	88	2780
Vision	303	90	2368
Visual search	292	98	2440
Visual-attention	253	93	1996
Memory	237	102	1855
Working memory	235	65	1772
Eye tracking	231	41	1320
Model	229	64	166
Search	219	68	1748
Movements	217	79	1552
Fixation	201	74	1516
Binocular vision	199	86	1415
Recognition	186	70	1457
Integration	185	97	1521

### Clustering function

Using the Clustering function, 13 groups were found, nine of which had a significant number of publications. The remaining four groups only made up 1.2% of the publications, which did not have enough publications to obtain connections between authors and publications.

Group 1 includes 692 publications and 2184 citations. The most-cited work is Itti & Koch (32) in Vision Research. This study describes a computerized implementation of the two-dimensional map that encodes the prominence or visibility of objects in the visual environment. That is, the competition between neurons means that there is a single winning location that corresponds to the next attended target. If this location is inhibited, the system will attend to the next most prominent location. This computational model combines stimulus-driven orientation, intensity, and colour information to address the extent to which the primate visual system can carry out visual search via one or more such saliency maps and how this can be tested.

**Figure 3.**
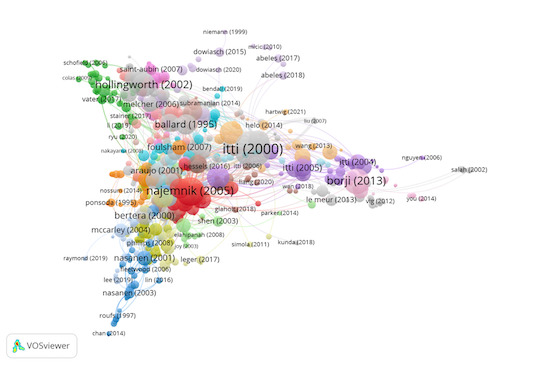
Citation network in Group 1

The scientific references in this group analyse the visual search procedure at the neurological level (Figure 3).

**Figure 4.**
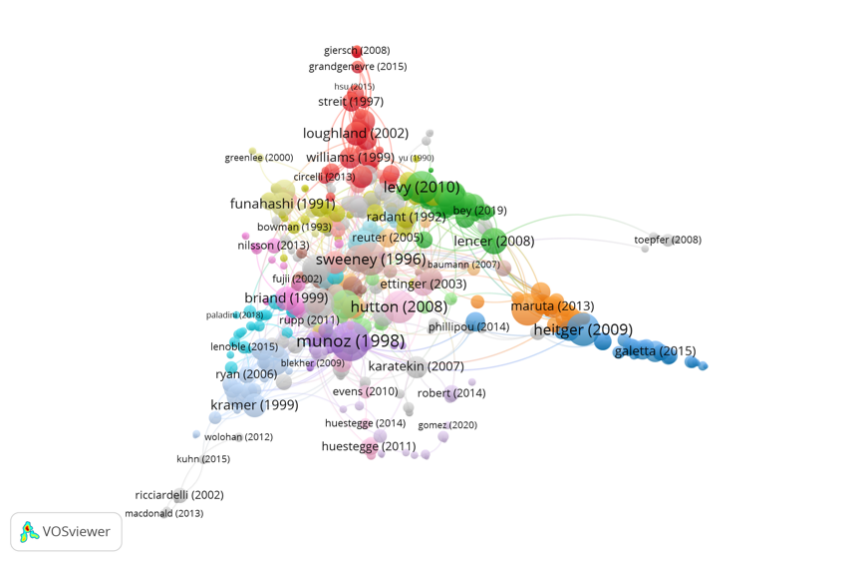
Citation network in Group 2

Group 2 comprises 536 publications and 1500 citations. The most-cited work is Munoz et al. (43) in Experimental Brain Research. This study suggests how saccadic movements change with age by quantifying the percentage of direction errors, the time to onset of the eye movement (saccadic reaction time: SRT) and the metrics and dynamics of the movement itself. The results show that children aged 5-8 years have very slow SRTs, but there is a great intra-subject variance in SRT, and, at the same time, they present the most direction errors in the anti-saccade task. Subjects aged 20-30 years have the fastest SRTs and lowest intra-subject variance in SRT. SRTs are slower and with longer saccades in subjects aged 60 to 79 years than other groups of subjects. This demonstrates very strong age-related changes, which can reflect different stages of normal development and degeneration of the nervous system. The improvement in performance in the anti-saccade task that occurs between the ages of 5 to 15 years is attributed to the maturation of the frontal lobes. The scientific references in this group analyse the neural mechanisms responsible for the generation of saccadic movements and how they change with age (Figure 4).

**Figure 5.**
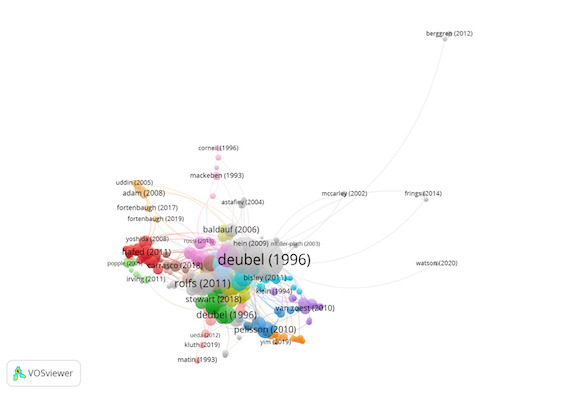
Citation network in Group 3

Group 3 includes 384 publications and 1209 citations. The most-cited work is Deubel & Schneider (15) in Vision Research, which also ranks first among the 20 most cited publications. The scientific references in this group analyse the association between saccadic eye movements and perceptual attention (Figure 5).

Group 4 comprises 264 publications and 910 citations. The most-cited work is Zihl (68) in Neuropsychologia. This study analyses eye movements in a group of patients suffering from homonymous hemianopia due to postgeniculate damage. After visual training, all patients show a significant improvement in visual searching (that is, the spatial organization of visual exploration was improved), indicating that a good oculomotor system can substitute the lost visual hemifield.

**Figure 6.**
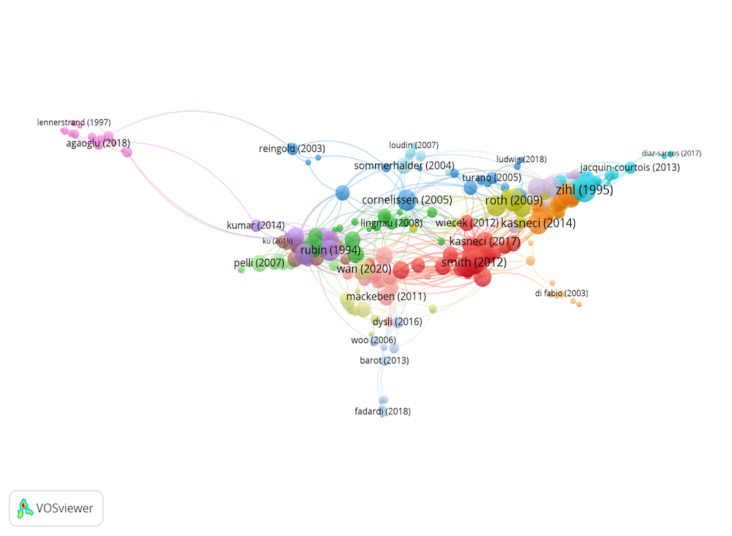
Citation network in Group 4

The scientific references in this group examine eye movements in patients with visual disturbances (Figure 6).

Group 5 includes 253 publications and 809 citations. The most-cited work is Savelsbergh et al. (54) in Journal of sports sciences.

This study analyses the skills of anticipation and visual search in expert and novice soccer goalkeepers. Expert goalkeepers are generally more accurate. That is, they use a more efficient search strategy involving fewer fixation. No differences in visual search behaviour are observed between successful and unsuccessful penalties.

**Figure 7.**
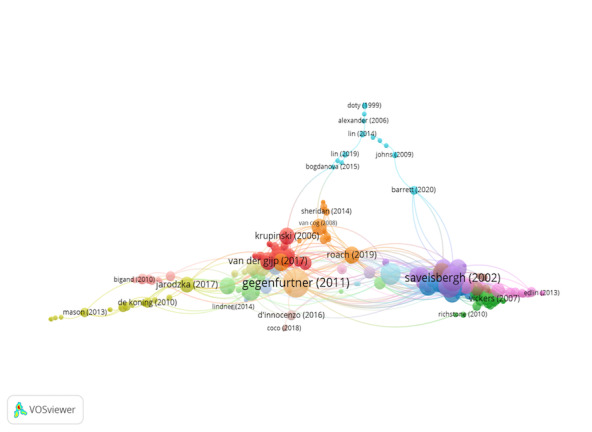
Citation network in Group 5

The scientific references in this group discuss the importance of oculomotor movements in sports (Figure 7).

Group 6 comprises 249 publications and 474 citations. The most-cited work is Konstantopoulos, Chapman & Crundall (36) in Accident analysis and prevention. This study examines the eye movements of driving instructors and learner drivers while they drive three virtual routes under different visibility conditions. The results show that eye movement strategies improve with driving experience. The high accident risk of night and rain driving could be partly explained by the decrement in visual search strategies during these conditions.

**Figure 8.**
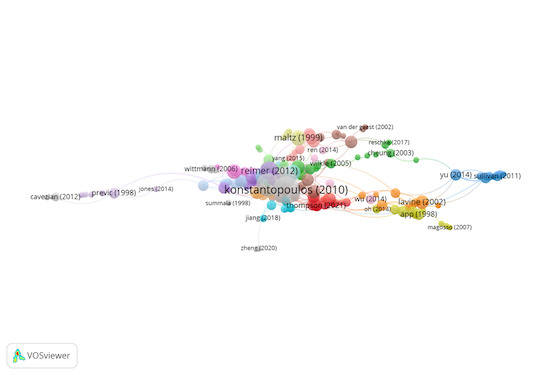
Citation network in Group 6

The scientific references in this group assess the importance of eye movements in driving (Figure 8).

Group 7 includes 232 publications and 477 citations. The most-cited work is Shadlen & Newsome (58) in Journal of Neurophysiology. The study aims to examine the activity of individual neurons in the posterior parietal cortex of two rhesus monkeys by discriminating the direction of movement on random point visual stimuli. The results show that stronger movement leads to larger neural responses early in the movement display period or when the direction of movement is towards the response field. However, greater suppression occurs, when the movement moves away from the response field. In this sense, individual neurons in the posterior parietal cortex display the information of gaze changes and the sensory information that instructs such a response. The time course of the neural response suggests that the posterior parietal cortex accumulates sensory signals relevant to the selection of a target for an eye movement.

**Figure 9.**
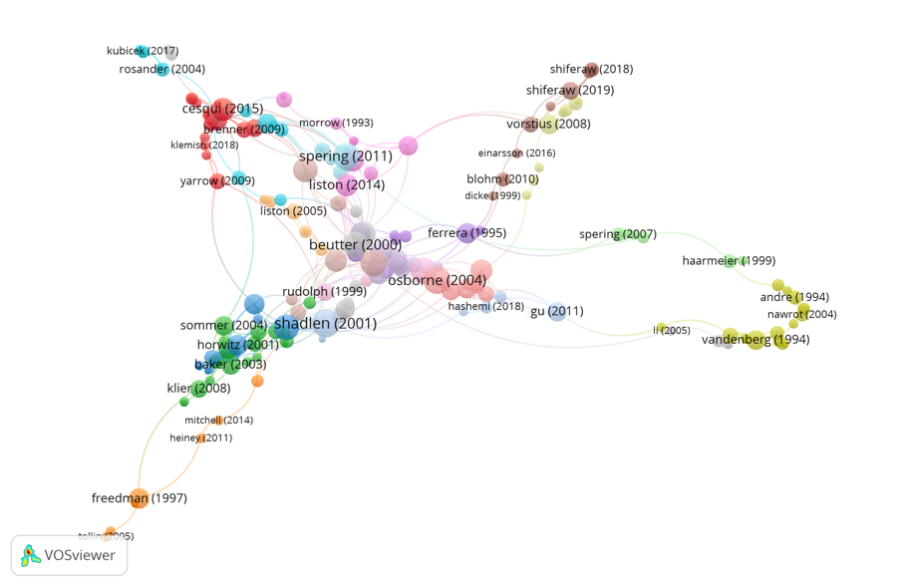
Citation network in Group 7

The scientific references in this group discuss the importance of cortical areas in eye movements, as well as changes in eye movements during sleep (Figure 9).

Group 8 comprises 188 publications and 444 citations. The most-cited work is Alvarez et al. (1) in Optometry and Vision Science. This research quantifies the clinical measurements and functional neuronal changes associated with vision therapy in patients with convergence insufficiency (CI). The findings show that the maximum speed of convergence is significantly slower in CI subjects compared to controls, which can result in asthenopic complaints in CI patients. Vision therapy can be associated with changes in clinical and cortical activity.

**Figure 10.**
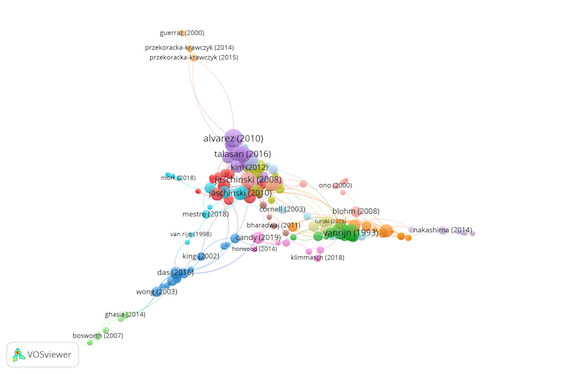
Citation network in Group

The scientific references in this group highlight the importance of eye movements in near vision tasks and their training using vision therapy (Figure 10).

Group 9 includes 175 publications and 322 citations. The most-cited work is Bosse & Valdois (13) in Journal of Research in Reading. This study focusses on the role of visual attention span on the development of reading skills in children. The results show that learning to read is influenced by the capacity for visual attention. In turn, visual attention span has a significant and sustained influence in all grades for irregular words. On the other hand, it is suggested that it could influence the acquisition of specific spelling knowledge over time.

**Figure 11.**
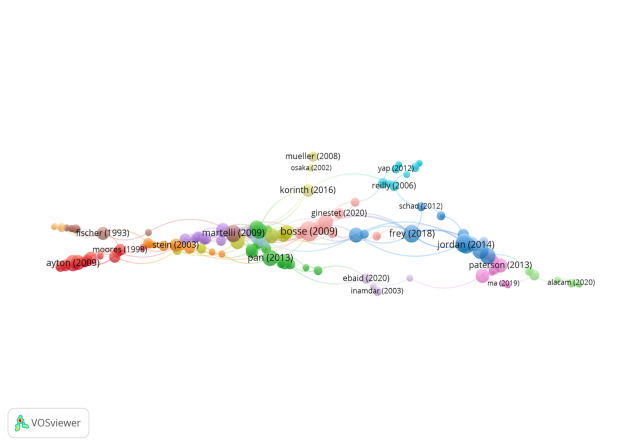
Citation network in Group 9

At a general level, the scientific references in this group describe the importance of eye movements in school performance (Figure 11).

### Core Function

1452 collected publications have four or more citations, representing 33% of the total citation network (7008). In other words, the field of eye movement research is multidisciplinary.

## Discussion

Major databases such as WOS or Scopus allow for the creation of citation networks. However, it is not possible to perform a systematic review of all of the existing scientific literature published on a topic, given that this does not provide a general overview of the connections between the citations of a group of publications. For this reason, the CitNetExplorer software was used, as, besides creating citation networks, it also offers a more detailed analysis of the scientific literature than the WOS or Scopus databases (62)

The general objective of the present study was to analyse the existing scientific literature on ocular motility. The source of publications was the WOS database, which only accepts prestigious international journals after a rigorous selection process, and whose search range begins in the year 1900.

Therefore, once the bibliography had been downloaded from the WOS database, the CitNetExplorer software allowed us to collect and analyse every available piece of literature on ocular motility from 1900 to May 2021. In addition, it was also possible to obtain the connections between the fields of study and the different research groups by analysing the citation networks. The "Clustering" function allowed us to obtain the results and group the publications according to the links between the citations. The "Core publications" function was used to select the most cited publications (that is, those studies with a minimum number of citations). Those publications with 4 or more citations were selected according to other studies (65). All these functions allow for a complete analysis of the publications related to the research field of interest (in this case, ocular motility).

Vision Research, with an impact factor of 2.61, was the journal with the highest number of publications (213) on ocular motility. In turn, the journal with the highest impact factor was The Journal of Neuroscience with 5.67. In any case, it is important to consider that the impact factor is a critical index of the journal’s importance but it is not an absolute index. The main difference between a critical index and an absolute index is that the latter is based on the impact of the research results and the authors’ physical and intellectual contributions (10).

However, the Journal of Eye Movement Research, since 2016, is the journal that has most increased its publications on eye movements and the impact they can have on academic performance (5; 14; 19; 59; 40). This may be because it is an eye movement-specific, peer-reviewed, open access journal devoted to all aspects of oculomotor functioning, including eye recording methodology, neurophysiological and cognitive models, attention, reading, as well as applications in neurology, ergonomics, media research. However, the article with the greatest impact was that of Kruger et al. (38), in which they compared the distribution of visual attention between subtitles and other sources of information through eye tracking and related this to academic comprehension and cognitive load measured through self-report questionnaires and electroencephalogram.

The country with the highest publication rate was the United States (33.9%), followed by England (14.9%) and Germany (13.1%). Our results agree with the study by Hernandez-Torre & Yuh-Shan (27). This suggests that these countries have a high interest in the influence of multiple factors at the academic level.

Among the first publications in this field of research, the articles by Schall (55), with a citation index of 19, and Ralph, Hager & Christine (50), with a citation index of 30, are particularly worth mentioning given the number of citations. In the first study, the authors used the adaptive tracking test, smooth pursuit analysis, saccadic eye movements, and visual analogue lines to analyze the effects of single oral doses of 5, 10, and 20 mg of Temazepam on eye movements. It was observed that Temazepam (20 mg) caused effects in all trials, with the maximum effect occurring after 30 minutes. The 10 mg dose affected saccadic eye movements, and the 5 mg dose was detected only by analyzing saccadic eye movements (55).

In Ralph, Hager & Christine (50), the authors corroborated the hypothesis that testing working memory beyond a certain threshold could result in decreased inhibition, with behavior resembling the errors made by patients with prefrontal dysfunction.

From 2013 to date, the curve of publications on eye movements has been steadily increasing, perhaps due to the increasing interest in the importance of vision on academic performance in recent years, and therefore the relevant role played by eye movements (46; 25; 57; 34; 67; 31). For example, Birch & Kelly (9) affirmed that reading is fundamental for the proper development of the learning process and good academic performance, emphasising the key role that saccades play in optimal reading development. Recently, Reddy et al. (53) used the ReadAlyzer Eye Movement recording system to conduct an objective assessment of eye movements on 30 subjects who presented with traumatic brain injury (TBI) and 60 control subjects. The TBI subjects showed a significant reduction in the measured parameters (fixations, regressions, reading rate, equivalent grade level, and reading comprehension) compared to those in the control group. Molina et al. (41) published a study in the American Academy of Optometry (AAO) in which the Visagraph Eye Movement recording system was used to analyse eye movement patterns in reading. In this case, 21 of the 41 subjects had attention deficit hyperactivity disorder (ADHD) and 20 were control subjects. All of the subjects orally read a standardised text that was suitable for their age group. The results showed that the ADHD subjects had worse eye movement patterns than the control group. These two studies analysed the quality of eye movements on reading in subjects with neurological disorders, with both obtaining similar results.

The year with the highest number of "key year" publications on eye movements was 2018. In this year, the study by Stewart & Schütz (60), which exposed how the presence of an attentional distractor affects integration performance both before saccadic movement onset and during saccadic movement execution is particularly worth mentioning. This study suggested that visual attention may be a mechanism for facilitating transsaccadic integration. Another publication by Ohl & Rolfs (48), concluded that saccadic movements exert spatially selective biases on stable representations in visuospatial working memory (VSWM).

Consequently, eye movements are present in all areas of daily life. Therefore, in recent years there has been an increasing amount of research conducted into the ways in which eye movements vary with age and how they influence memory (28; 11; 56). Image encoding is related to eye movements and declines in spatial memory may be associated with a specific decline in spatial processing rather than general age-related declines in terms of cognition. However, the reduction in learning ability does not appear to be affected by changes in the control of visual attention through eye-tracking, nor by changes in attentional engagement.

Another topic of interest, although to a lesser extent, is the importance of eye movements in sports vision (47; 63). It has been observed that elite players present a better visual search strategy than mid-level players, therefore suggesting that the position and situation of teammates and opponents are relevant sources of information that enable players to make accurate and consistent passing decisions.

In conclusion, by analyzing citation networks it has been possible to extend knowledge about eye movements. Nine groups were identified in this study (age, sports vision, school performance, visual field disturbances, visual therapy, sleep, neurology, driving, and perceptual attention), therefore meaning that the field of research on eye movements is multidisciplinary. The topic with the most publications to date is the visual search procedure at the neurological level. However, research topics on the importance of eye movements with age and sports vision have gained growing interest in recent years.

### Ethics and Conflict of Interest

The author(s) declare(s) that the contents of the article are in agreement with the ethics described in http://biblio.unibe.ch/portale/elibrary/BOP/jemr/ethics.html and that there is no conflict of interest regarding the publication of this paper.
